# Evaluation of fusion protein cleavage site sequences of Newcastle disease virus in genotype matched vaccines

**DOI:** 10.1371/journal.pone.0173965

**Published:** 2017-03-24

**Authors:** Shin-Hee Kim, Zongyan Chen, Asuka Yoshida, Anandan Paldurai, Sa Xiao, Siba K. Samal

**Affiliations:** Virginia-Maryland Regional College of Veterinary Medicine, University of Maryland, Greenmead Drive, College Park, Maryland, United States of America; Korea University College of Medicine and School of Medicine, REPUBLIC OF KOREA

## Abstract

Newcastle disease virus (NDV) causes a devastating poultry disease worldwide. Frequent outbreaks of NDV in chickens vaccinated with conventional live vaccines suggest a need to develop new vaccines that are genetically matched against circulating NDV strains, such as the genotype V virulent strains currently circulating in Mexico and Central America. In this study, a reverse genetics system was developed for the virulent NDV strain Mexico/01/10 strain and used to generate highly attenuated vaccine candidates by individually modifying the cleavage site sequence of fusion (F) protein. The cleavage site sequence of parental virus was individually changed to those of the avirulent NDV strain LaSota and other serotypes of avian paramyxoviruses (APMV serotype-2, -3, -4, -6, -7, -8, and -9). In general, these mutations affected cell-to-cell fusion activity *in vitro* and the efficiency of the F protein cleavage and made recombinant Mexico/01/10 (rMex) virus highly attenuated in chickens. When chickens were immunized with the rMex mutant viruses and challenged with the virulent parent virus, there was reduced challenge virus shedding compared to birds immunized with the heterologous vaccine strain LaSota. Among the vaccine candidates, rMex containing the cleavage site sequence of APMV-2 induced the highest neutralizing antibody titer and completely protected chickens from challenge virus shedding. These results show the role of the F protein cleavage site sequence of each APMV type in generating genotype V-matched vaccines and the efficacy of matched vaccine strains to provide better protection against NDV strains currently circulating in Mexico.

## Introduction

Virulent strains of Newcastle disease virus (NDV) cause a devastating disease in chickens leading to major economic losses in poultry industry worldwide [1]. NDV belongs to the genus *Avulavirus* in the family *Paramyxoviridae*. The single-stranded negative-sense RNA genome of NDV contains six transcriptional units (3′-N-P-M-F-HN-L-5′) [2]. The fusion (F) and hemagglutinin-neuraminidase (HN) proteins are surface glycoproteins and protective antigens of NDV. The F protein directs the viral fusion activity by interacting with the HN protein [2]. The F protein cleavage site sequence is a major determinant of NDV virulence [3, 4].

NDV strains are classified into three different pathotypes: highly virulent (velogenic), intermediate (mesogenic), or avirulent (lentogenic) [1]. All NDV strains belong to one serotype, but there is antigenic and genetic diversity among strains [5, 6]. NDV strains have been classified into two major classes on the basis of genome length and the sequence of the F gene. The class I strains are mostly avirulent and isolated from wild birds. Class II strains contain both virulent and avirulent viruses that have been recovered from wild and domestic birds and are further divided into 18 genotypes [7]. In general, the most prevalently circulating genotypes are V, VI and VII of class II NDV strains [5].

Newcastle disease (ND) is one of the major diseases of poultry in the world, despite the vaccination of poultry with lentogenic NDV strains, indicating inefficacy of currently used vaccines. The currently used vaccine strains, such as B1 and LaSota (genotype II), were developed in 1940’s in the U.S. They are 21–23% genetically different from the viruses currently circulating in different parts of the worlds. In fact, several recent studies showed that both inactivated and live attenuated vaccines developed from currently circulating genotype strains provided better protection than the conventional vaccines. [8–14].

In Mexico, the outbreaks of ND in the poultry industry have occurred despite the use of vaccines. The virulent NDV strains circulating in Mexico are predominantly genotype V [2]. Our sequence analysis showed that the percent amino acid sequence identity of the F and HN proteins between NDV strain Mexico/01/10 and the currently used vaccine strain LaSota (genotype II) is 89% [15]. This indicates that the circulating strains are substantially divergent from the vaccine strain in use and suggests that antigenic differences probably contribute to poor vaccine protection [10, 16]. Therefore, in this study, we have generated genotype-matched vaccine candidates by a reverse genetics technique. The vaccine virus candidates were derived from NDV strain Mexico/01/10 by changing its F protein cleavage site sequence to those of the avirulent strain LaSota and other avian paramyxovirus (APMV) types. Various recombinant Mexico/01/10 (rMex) derivatives (genotype V) were compared to strain LaSota (genotype II) for replication *in vitro* and as live vaccines for immunogenicity and protective efficacy against challenge with homologous virulent NDV strain Mexico/01/10 (genotype V). Most rMex mutant viruses were more effective than the vaccine strain LaSota in preventing the shedding of virulent NDV strain Mexico/01/10 in chickens, indicating the superior efficacy of genotype-matched vaccines.

## Materials and methods

### Reverse genetics

**Fig 1:**
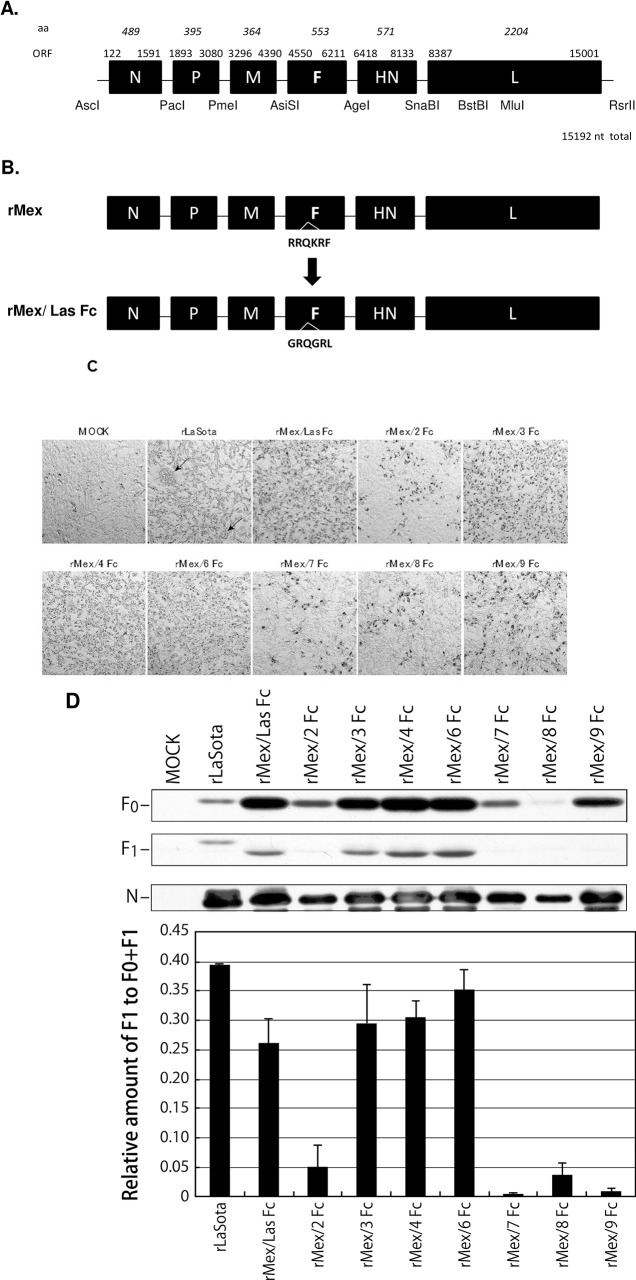
Genome map of NDV Mexico/01/10 (wtMex) and *in vitro* characterization of its recombinant derivatives. (A) Genome map of wtMex, with amino acid lengths indicated in italics above the map and location of each ORF (upper diagram) and strategy of construction of a complete antigenome cDNA encoding recombinant Mex (rMex), with unique restriction sites noted (lower diagram). (B) Modification of the F protein cleavage site of rMex from RRQKR/F to GRQGR/L, yielding rMex/ Las Fc. (C) For evaluating production of syncytia, DF1 cells in six-well plates were infected with rLaSota or each rMex mutant virus at a multiplicity of infection (MOI) of 0.01, incubated for 72 h in the presence chicken egg allantoic fluid as a source of added protease, and visualized by immunoperoxidase staining using antiserum against the N protein of NDV, with viral antigen stained red. (D) Western blot analysis of proteolytic cleavage of the F0 proteins of rLaSota and rMex mutant viruses in infected DF1 cells that were infected at an MOI of 0.1, incubated in the presence of added allantoic fluid, harvested 24 hpi, and visualized with anti-NDV rabbit antiserum. The positions of the precursor protein F_0_ and the cleavage product F_1_ are indicated. The relative levels of the F_0_ and F_1_ proteins in the Western blot experiment were measured by Bio-Rad Gel Image analysis, and the efficiency of cleavage was determined by dividing the amount of F_1_ by the amount of F_1_ plus F_0_. Each bar represents mean and standard error of the mean of triplicate samples.

NDV strain Mexico/01/10 (wtMex) was isolated from an outbreak of ND in a commercial poultry in Mexico [15]. For the construct, viral RNA was isolated from NDV-infected embryonated chicken eggs, and eight fragments were generated by reverse transcription-PCR (RT-PCR) (Fig 1A). A full-length cDNA of the complete 15,192-nt-long antigenome of wtMex was constructed in plasmid pBR322/dr using unique restriction enzyme sites. For generation of vaccine candidates, the F protein cleavage site was mutated using overlapping PCR and the mutated sequence for each virus is listed in Table 1. Infectious recombinant viruses were recovered as previously described procedure for NDV [17]. The presence of unique restriction enzyme sites and the sequences of the F protein cleavage sites in the recovered viruses were confirmed by RT-PCR analysis.

**Table 1:** Fusion protein cleavage site sequence of rMex mutant viruses and their pathogenicity in 1-day-old chickens.

Virus	Strain^a^	F protein cleavage site	ICPI^b^
rLaSota	LaSota	G**R**QG**R**↓L	0.00
rMex/ Las Fc	LaSota	G**R**QG**R**↓L	0.00
rMex/ 2 Fc	Yucaipa	**K**PAS**R**↓F	0.00
rMex/ 3 Fc	Netherlands	**R**P**R**G**R**↓L	0.00
rMex/ 4 Fc	Hong Kong	DIQP**R**↓F	0.00
rMex/ 6 Fc	Hong Kong	APEP**R**↓L	0.00
rMex/ 7 Fc	Tennessee	LPSS**R**↓F	0.00
rMex/ 8 Fc	Delaware	YPQT**R**↓L	0.00
rMex/ 9 Fc	New York	I**R**EG**R**↓I	0.00

^a^Each cleavage site sequence of fusion protein was originated from the listed strain of avian paramyxoviruse serotypes.

^b^Intracerbral pathogenicity index (ICPI): evaluation of disease and death following intracerebral inoculation in 1-day-old SPF chicks. Pathotype definition: virulent strains, 1.5–2.0; intermediate virulent strains, 0.7–1.5; and avirulent strains, 0.0–0.7.

### *In vitro* growth characterization and pathogenicity of recombinant vaccine viruses

The multicycle growth kinetics of rLaSota and vaccine viruses was evaluated in DF1 cells in the presence of 10% chicken egg allantoic fluid. The ability of the vaccine virus to form syncytia and plaques was characterized by infecting DF1 cells with rLaSota or rMex/AF (MOI of 0.01) in the presence or absence of 10% normal allantoic fluid. Cleavage efficiency of the F proteins of vaccine viruses was evaluated by Western blot analysis with anti-NDV F rabbit polyclonal antiserum. Pathogenicity of rMex mutant viruses was evaluated by the intracerebral pathogenicity index (ICPI) assay in 1-day-old chicks [1]. Genetic stability of vaccine viruses was evaluated by *in vivo* passage in the respiratory tract of 1-day old chicks using an oculonasal infection [14]. After the fifth passage, the viral genome RNA was isolated from trachea and RT-PCR was performed for the sequence analysis.

### Challenge of immunized chickens with highly virulent NDV strains

The protective efficacy of vaccine candidates was evaluated in 2-week-old chickens. SPF chickens (10 birds for each group) were immunized with vaccine viruses (200 μl of each, 10^6^ EID_50_) by the oculonasal route. One group of 3 chickens remained as unvaccinated controls. The chickens were transferred into a USDA-certified BSL-3 containment facility, and at 3 weeks post -immunization (wpi), birds were challenged with 100 CLD_50_ (chicken 50% lethal dose) of virulent NDV strain Mexico/01/10 through the oculo-nasal route [14]. The chickens were observed daily for 10 days for clinical signs and mortality. Shedding of challenge virus was evaluated by collecting oral and cloacal swabs from birds at 4 and 7 days post-challenge (dpc). The presence of virus was determined by inoculating clarified swab samples into 9-day-old SPF embryonated chicken eggs and conducting HA assays 3 days later. Serum samples were collected at day 0 prior to vaccination and at day 21 post-vaccination. Serum antibody titers were determined by hemagglutination inhibition (HI) assay using rLaSota or rMex/AF as an antigen. All animal experiments were approved by the committee of IACUC, University of Maryland (protocol number R-15-80). Animal care and handling, including euthanasia were conducted following the guidelines of the American Veterinary Medical Association. All experiments involving virulent strains of NDV were performed in our USDA approved enhanced Biosafety Level-3 facility.

## Results

### Generation of live-attenuated vaccine candidates

To develop the reverse genetics of NDV strain Mexico/01/10, the cDNA segments generated from viral RNA were cloned in a sequential manner into the low-copy-number plasmid pBR322/dr [17] between a T7 promoter (TAATACGACTCACTATAGG) and the hepatitis delta virus ribozyme sequence (Fig 1A). To generate vaccine viruses, the F protein cleavage site of Mexico/01/10 (RRQKR↓F) was first replaced with a cleavage site that is common in avirulent strains, including B1 and LaSota: GRQGR↓L (Fig 1B). Similarly, its cleavage site was changed with those of seven different APMV serotypes (Table 1). All these APMVs are avirulent and their cleavage site sequences are lacking a polybasic furin motif (Arg-X-Arg/Lys-Arg↓) [2]. The recombinant Mexico/01/10 and mutant viruses were readily recovered following the standard protocol for NDV [17]. The HA positive allantoic fluid was processed to isolate viral genomic RNA for RT-PCR and sequence analysis of the F gene, which confirmed the correct sequence of the gene and the F protein cleavage site.

We further evaluated whether the mutation of the F cleavage site altered the ability of the virus to form syncytia and plaques in cell culture. The parental Mexico/01/10 virus produced extensive syncytia and large plaques with or without exogenous protease (data not shown), which is typical of virulent NDV strains. In contrast, rMex mutant viruses did not cause the formation of syncytia or plaques in the absence or presence of added protease (data not shown). Immunostaining of the infected cells confirmed single-cell infections caused by rMex mutant viruses in the presence or absence of added protease, whereas strain LaSota produced single-cell infections in the absence of protease and syncytial infections in the presence of protease (Fig 1C). Further, Western blot analysis confirmed that the cleavage of F protein of mutant viruses rMex/2Fc, rMex/7Fc rMex/8Fc, and rMex/9Fc was less efficient than that of rLaSota (p<0.05) (Fig 1D).

**Fig 2:**
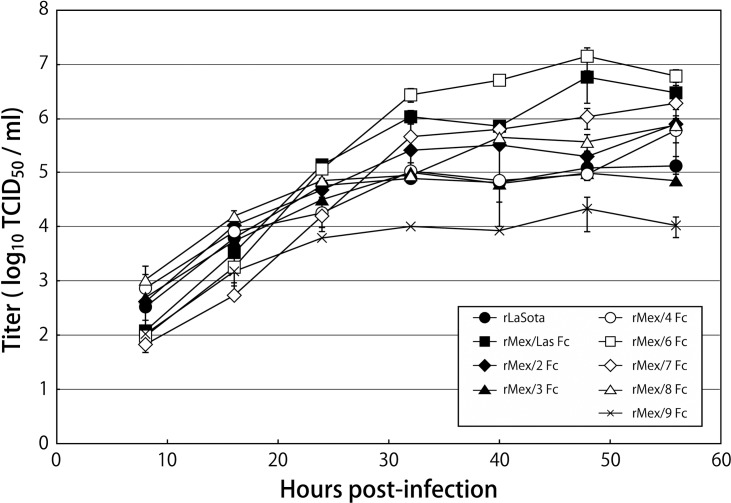
Growth kinetics of LaSota and rMex mutant viruses in chicken embryo fibroblast DF1 cells following infection with an MOI of 0.01 PFU/cell. Uninfected chicken egg allantoic fluid was included as a source of exogenous protease. The viral titers were determined by limiting dilution on DF1 cells.

To evaluate the potential of these mutant viruses as vaccine candidates for NDV, *in vitro* replication of rMex mutant viruses was compared with that of LaSota vaccine virus in DF1 cells. In general, multi-cycle growth kinetics of the viruses did not show much significant difference, indicating the comparable replication of rMex mutant viruses to rLaSota (Fig 2). Interestingly, only rMex/ 7Fc reached significantly higher titers than rLaSota at 40 hpi and 56 hpi (p<0.05). These results indicated that inefficient cleavage of F protein did not affect the replication of these mutant viruses, but the NDV internal proteins could contribute to the efficient replication of the viruses *in vitro*.

The pathogenicity of rMex mutant viruses was evaluated by the intracerebral pathogenicity index (ICPI) test in 1-day-old SPF chicks. The ICPI values of wtMex and rMex viruses were 1.89 and 1.88, respectively, which are within the range of 1.5 to 2.0 that defines the virulent phenotype (data not shown). In contrast, all the rMex vaccine viruses were highly attenuated, with an ICPI value of 0.00 that defines the lentogenic phenotype (Table 1). None of the chicks infected with the rMex mutant viruses had any clinical signs or mortality during 8 days of observation.

We further evaluated whether the attenuated rMex mutant viruses could revert back to wild-type virus *in vivo* by passaging each virus five times in 1-day-old chicks via the oculonasal route. After the final passage, the recovered virus was subjected to RT-PCR and sequence analysis of the F protein (data not shown). The sequence did not show reversion or any other mutations, suggesting the genetic stability of the rMex mutant viruses in chickens. Our results showed that the mutation of F protein cleavage site sequence rendered stable live-attenuated vaccine candidates representing circulating Mexican NDV strains.

### Immunogenicity and protective efficacy of rMex vaccine viruses

**Fig 3:**
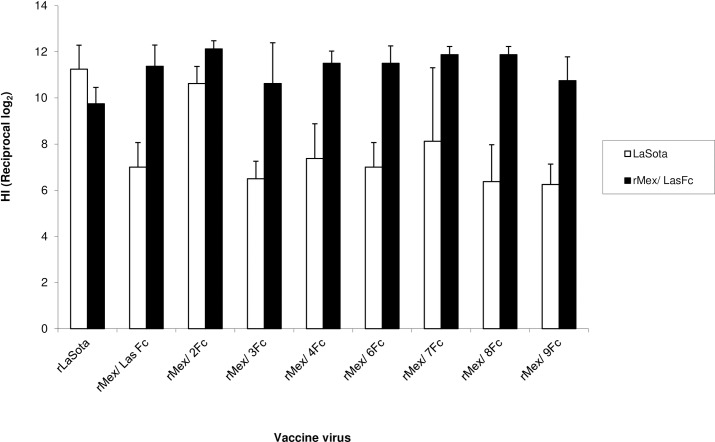
Induction of virus-specific serum antibodies in 2-week-old chickens in response to oculo-nasal infection with rLaSota and rMex mutant viruses. Chickens were inoculated with each virus (indicated on the x-axis) by the oculo-nasal route, mimicking natural infection. Sera were collected at 3 weeks post-infection. Virus-specific antibodies were measured by a hemagglutination inhibition assay using rLaSota or rMex/Las Fc virus and chicken erythrocytes.

To evaluate protective immunity, two-week-old chickens in nine groups of 10 each were immunized with rMex mutant viruses or rLaSota by the oculo-nasal route. The serological cross-reaction between rMex mutant viruses (genotype V) and rLaSota (genotpe II) was evaluated by collecting serum samples at 3 wpi for hemagglutination-inhibition (HI) assay against rLaSota or rMex/ Las Fc virus. All pre-vaccination sera were negative to NDV by the HI test (data not shown). In the rLaSota-specific assay, antisera raised against rLaSota had higher HI titers than those raised against rMex mutant viruses (p<0.05) (Fig 3). Only rMex/ 2Fc induced comparable level of immune response to rLaSota. In contrast, in the rMex mutant virus-specific assay, most of antisera raised against rMex mutant viruses had higher titers than those against rLaSota (p<0.05) (Fig 3). Two groups of sera raised against rMex/ 3Fc and rMex/ 9Fc showed the similar levels of antibody response to that against rLaSota. Overall, our result showed that rMex/ 2Fc induced good antibody response against both homologous and heterologous antigens.

To evaluate protective efficacy the chickens in each immunization group were challenged 3 wpi with 100 CLD_50_ per chicken of the virulent wtMex via the oculo-nasal route. For the unimmunized chickens (three chickens), challenge with highly virulent NDVs resulted in 100% of mortality at 4 day post-challenge (dpc). All of immunized chickens were fully protected from clinical disease and mortality against wtMex (data not shown). The shedding of challenge viruses was evaluated from oral and cloaca swabs at 4 dpc and 7 dpc (Table 2). In birds immunized with LaSota vaccine (genotype II), there was challenge virus breakthrough at four anatomical sites among the 10 birds for the heterologous rMex challenge (genotype V). In homologous challenge groups, different cleavage site sequences affected protective efficacy of rMex vaccine candidates. For birds immunized with the rMex/ 2Fc and rMex 8Fc, the chickens were completely protected from challenge virus shedding (Table 2). The rest of the mutant viruses provided incomplete protection from virus shedding. On day 7, shedding of challenge viruses was not detected in any immunized birds. Our overall results indicated that the sequence of F protein cleavage site may play an important role in the conformation of the F protein, thus modulating the immune responses and the protective efficacy of vaccine virus.

**Table 2:** Oral and cloacal shedding of challenge NDV strain Mexico/01/10.

	4 days post-challenge		7 days post-challenge	
Vaccine virus^a^	Oral	Cloacal	Oral	Cloacal
rLaSota	2/10	2/10	0/10	0/10
rMex/Las Fc	1/10	0/10	0/10	0/10
rMex/ 2 Fc	0/10	0/10	0/10	0/10
rMex/ 3 Fc	2/10	0/10	0/10	0/10
rMex/ 4 Fc	0/10	1/10	0/10	0/10
rMex/ 6 Fc	0/10	1/10	0/10	0/10
rMex/ 7 Fc	0/10	1/10	0/10	0/10
rMex/ 8 Fc	0/10	0/10	0/10	0/10
rMex/ 9 Fc	0/10	1/10	0/10	0/10

^a^Groups of 2-week-old chickens were inoculated with each virus by the oculo-nasal route and challenged with NDV strain Mexico/01/10. Oral (A) and cloacal (B) swabs were collected from the 10 birds in each group on day 4 and 7 post challenge. To confirm the shedding of challenge virus, aliquots (100 μl each, out of a total of 1 ml of swab fluid) of the collected samples were inoculated into three eggs, and allantoic fluids were collected on 3 dpi. Virus replication was determined by hemagglutination assay.

## Discussion

ND is enzootic in many countries [5]. Despite the vaccination with strains B1 and LaSota, there have been numerous outbreaks of ND in vaccinated chickens in different parts of the world [18, 19]. Recent studies have shown that NDV strains currently in circulation represent genotypes that differ from that of the vaccine strains (genotype II) [5, 20]. The genotype-matched vaccines of currently circulating genotype VII strains have shown enhanced effectiveness in preventing virus shedding [10, 16] and providing protection [13, 14], highlighting a need to develop genetically matched vaccines against NDV strains currently circulating in different geographic regions.

In Mexico and Central America, ND outbreaks occur frequently. The most commonly isolated NDV strains in these countries belong to the genotype V [21, 22]. However, a lack of reverse genetics for genotype V has hindered the development of efficient vaccine for a better control of NDV. Therefore, in this study, we generated a reverse genetics system for NDV strain Mexico/01/10 for development of an attenuated vaccine strain. For vaccine development, the F protein cleavage site sequence of the virus was modified into that of avirulent NDV strain LaSota, since the proteolytic cleavage is a major determinant of NDV virulence [4]. This approach has provided a very simple and rapid means of producing attenuated vaccines for genotype VII [13, 14]. In this study, its cleavage site was further mutated to avirulent cleavage site sequences of other APMV types to generate the most effective vaccine for genotype V.

*In vitro* comparison of the rMex mutant viruses with the conventional vaccine strain LaSota indicated that vaccine viruses can replicate efficiently in the presence of added allantoic fluid as a source of protease. However, the rMex mutant viruses caused single cell infection without syncytium or plaque formation in DF1 cells in the presence of exogenous protease. Several rMex mutant viruses showed significantly inefficient cleavage/activation compared to LaSota. This also has been observed previously with the F protein of virulent genotype VII NDV [14]. These results suggest that, in addition to the cleavage site sequence, other regions in the F protein influence cleavage activation and possibly fusion activity, perhaps by affecting F protein conformation. Nonetheless, most of rMex mutant viruses were able to replicate efficiently in *vitro* and *in vivo*, suggesting that lack of cell-to-cell fusion activity did not affect virus replication.

In generating a safe vaccine, reversion of a mutated F protein cleavage site to a wild-type sequence has been a major concern [23]. In this study, the genetic stability of rMex mutant viruses was confirmed by five passages by the natural route of infection in 1-day-old chicks. We did not detect any changes in sequence or clinical signs, suggesting the generation of stably attenuated vaccine viruses.

The efficacy of vaccine in the field can be affected by poor vaccination practices, field environmental and/or immunosuppressive factors [19]. However, the failure of the vaccination leading to the recurrent outbreaks of ND can attribute to antigenic mismatch between the genotype II commercial vaccine strains and the currently circulating virulent strains. We previously demonstrated complete protection of chickens by immunization with genotype VII-matched vaccines generated either by chimeric or full-genome approach [14, 24]. In the present study, immunization with rLaSota or Mex/AF was substantially effective against challenge with virulent NDV strains, but the homologous challenge was more restricted than the heterologous challenge. In this particular study, in a laboratory setting, complete protection against visible disease was achieved for both homologous and heterologous challenge. However, vaccination under field conditions often is less effective than in the laboratory setting, and it seems likely that the homologous immunization would be more protective in the field.

Our findings indicate that reverse genetics can be used to rapidly develop a live-attenuated vaccine based on currently circulating virus, and that the protection efficacy of the vaccine can be improved by using genotype-matched vaccine viruses. Specifically, our immunogenicity and challenge study showed that modification of the cleavage site sequence to that of rLaSota may not be the best approach to generate an effective vaccine for NDV genotype V. Instead, the cleavage site sequence of APMV-2 can be a better choice for efficacy of genotype V vaccine. This study also needs to be verified by evaluating protective efficacy in broiler chickens.
