# Expression of Concern: Evaluation of fusion protein cleavage site sequences of Newcastle disease virus in genotype matched vaccines

**DOI:** 10.1371/journal.pone.0244011

**Published:** 2020-12-09

**Authors:** The PLOS ONE Editors

After this article [1] was published, it came to light that the Data Availability Statement is inaccurate: the underlying data are not included with the published article. Additionally, there are six statements in the Results that rely on data not shown which is not allowable per the journal’s Data Availability Policy.

The authors clarified that the underlying data for this study are not available, and so the article does not comply with *PLOS ONE*’s Data Availability Policy.

The *PLOS ONE* Editors issue this Expression of Concern to notify readers of this issue. We regret that this was not addressed prior to publication.
